# Polymeric Systems as Antimicrobial or Antifouling Agents

**DOI:** 10.3390/ijms20194866

**Published:** 2019-09-30

**Authors:** Iolanda Francolini, Antonella Piozzi

**Affiliations:** Department of Chemistry, Sapienza University of Rome, P.le Aldo Moro, 5-00185 Rome, Italy

The rapid increase in the emergence of antibiotic-resistant bacterial strains combined with a dwindling rate of discovery of novel antibiotic molecules has lately created an alarming issue worldwide [1]. Resistant genes in microorganisms may be inherited from forerunners or acquired through genetic mutations or gene exchange [2]. Although the occurrence of resistance in microbes is a natural process, the overuse of antibiotics is known to improve the rate of resistance evolution [3]. Indeed, under antibiotic treatment, susceptible bacteria inevitably die, while resistant microorganisms proliferate under reduced competition. Therefore, the out-of-control use of antibiotics causes the elimination of drug-susceptible species that would naturally limit the expansion of resistant ones. On top of that, the ability of many microbial species to grow as biofilm has further complicated the treatment of infections with conventional antibiotics. Indeed, microbial biofilms, that is microbial communities growing attached to abiotic surfaces (medical devices, surgical instruments, industrial pipelines, etc.) and tissues [4], are known to be an optimal environment to amplify both naturally occurring and induced antibiotic-resistance phenomena [5]. That together with other defense mechanisms significantly increases biofilm antibiotic tolerance.

A number of corrective measures are currently under exploration to reverse or slow down antibiotic resistance evolution, among which the development of polymer-based antimicrobial compounds has emerged as one of the most promising solutions [6,7]. Indeed, antimicrobial polymers benefit from a non-specific mode of action, primarily targeting the microbial membrane, and generally display less propensity to promote antimicrobial resistance. Most of the so far investigated polymeric biocides are able to interact with the bacterial cell membrane causing membrane disassembly and leakage of intracellular material [8,9]. Interestingly, some antimicrobial polymers have also been reported to potentiate the activity of conventional antibiotics [10]. 

A plethora of different polymer systems has been designed to prevent or treat biofilm formation, including: (i) cationic polymers [11,12]; (ii) antibacterial peptide-mimetic polymers [13,14]; (iii) polymers or composites able to load and release bioactive molecules [15,16,17]; and (iv) antifouling polymers, able to repel microbes by physical or chemical mechanisms [18]. The potential fields of application of antimicrobial polymers are numerous. They may play a predominant role in the design and fabrication of medical devices as well as in food packaging and as drug carriers.

This special issue collected nineteen papers, of which four were reviews and fifteen were original articles. All of the four reviews were essentially focused on the application of antimicrobial polymers in the biomedical field [19,20,21,22]. The review by Cattò and Cappitelli [19] provided an overview of the most common methods for testing the antibiofilm activity of polymeric surfaces. The authors underlined how there is a general lack of standardized *in vitro* methods as well as controlled *in vivo* studies, which may question the relevance of obtained results. In this regard, simplified guidelines were proposed in the review to help readers choose the most appropriate tests for their objectives. 

The review by González-Henríquez and colleagues was instead focused on the manufacturing of 3D-printed objects based on antimicrobial polymers for the production of personalized devices, including implants and drug dosage forms [20]. In the first part of the review, a particular manufacturing technology to produce 3D-objects, that is “additive manufacturing”, was described, and illustrative examples of fabrication of 3D-objects using natural and synthetic antimicrobial polymers were discussed. 

The potentiality of antimicrobial polymers to replace existing antibiotics was reviewed by Kamaruzzaman et al. [21], who provided the latest updates in the context of ESKAPE (*Enterecoccus faecium*, *Staphylococcus aureus*, *Klebsiella pneumoniae*, *Acinetobacter baumannii*, *Pseudomonas aeruginosa*, and *Enterobacter* spp.) pathogens. Finally, the state of the art of antibacterial polymers against periodontal pathogens was reviewed by Chi et al. [22], who paid particular attention to polymeric systems for functional guided tissue regeneration (GTR) membrane, polymer composites for decay restoration, and photosensitizer (PS) modification for photodynamic therapy enhancement.

As for the 15 original articles of this special issue, they can be sketchily divided in two broad categories, namely studies focused on polymers able to kill microorganisms (*antimicrobial system*s) and studies focused on materials with fouling resistance properties (*antifouling systems*). 

Among the antimicrobial systems, readers can find antimicrobial peptides [23,24,25,26], cationic polymers [27,28,29,30,31], and inorganic/polymer composites [32,33,34]. 

Basically, four antimicrobial peptides were investigated: (i) Halictine-1, for which the correlation between changes in primary/secondary structure and antimicrobial activity was studied through various membrane-mimicking models [23]; (ii) Magainin 2, whose antibiofilm activity was tested against *Acitenobacter baumannii* strains [24]; (iii) the lipopeptide (C_10_)_2_-KKKK-NH_2_, whose potentiality, alone and in combination with lens liquids, in the prophylaxis of contact lens-related eye infections was studied [25]; and iv) a bactenecin-derivative peptide named 1018K6, which was conjugated to polyethylene terephthalate (PET) to obtain an active packaging for the food industry [26].

As far as cationic polymers are concerned, a thermally stable cationic polymer biocide was obtained by Moshynets and colleagues [27], through polymerization of guanidine hydrochloride and hexamethylenediamine. Such polymer biocide was then incorporated into Polyamide 11 film to obtain contact-active composites. Interesting antibiofilm activities were found against two biofilm-forming model bacterial strains, *E. coli* K12 and *S. aureus* ATCC 25923 [27]. 

The synthesis of peptides-mimicking amphiphilic cationic copolymers based on maleic anhydride and 4-methyl-1-pentene was reported by Szkudlarek et al. [28]. The copolymers were then quaternized with either methyl iodide or dodecyl iodide to stabilize polymer cationic charges. Of particular relevance was the minimum inhibitory concentration (MIC) of quaternized copolymers, which was found to be lower than Nisin on a molar basis. 

Cationic acrylic copolymers based on poly(2-hydroxy ethyl methacrylate) (HEMA), a largely employed biocompatible polymer, were investigated in 2 of the 15 studies of this special issue [29,30]. Specifically, Muñoz-Bonilla et al. [29] copolymerized HEMA with a methacrylic monomer bearing a thiazole side group susceptible to quaternization, while Galiano et al. [30] used UV-induced polymerization to copolymerize HEMA with two cationic acryloyloxyalkyltriethylammonium bromides (C-11 or C-12 alkyl chain linker). In both studies, copolymers exhibited significant activity versus Gram-positive (*S. aureus*) and Gram-negative (*P. aeruginosa* and *E. coli*) bacteria and, as expected, copolymer antimicrobial activity increased with increasing of the cationic unit content. Cationic poly(methylmethacrylate)-based nanoparticles were instead prepared by Galvão et al. [31]. The layering of such nanoparticles onto model surfaces (silicon wafers, glass, and polystyrene sheets) resulted in a significant reduction (ca. 7 logs) of the number of *E. coli* and *S. aureus* adhered onto the coated-surfaces compared to pristine surfaces.

Always in the framework of antimicrobial systems, three types of antibacterial inorganic/polymer composites were reported in this special issue [32,33,34]. Antibacterial cuprous oxide nanoparticles (Cu_2_ONPs) were loaded into linear low-density polyethylene (LLDPE) by Gurianov et al. [32] to develop materials for tap water and wastewater disinfection. Inorganic silica materials functionalized with various types of organic groups (3-aminopropyl, 3-mercaptopropyl, or 3-glycidyloxypropyl groups) were used as bone-targeted delivery systems for metronidazole [33]. Antibacterial and antioxidant phenol molecules, extracted from olive mill wastewater, were intercalated into the host structure of ZnAl layered double hydroxide and employed for the preparation of poly(butylene succinate) composites by Sisti et al. [34]. These composites showed interesting properties for application in food packaging. 

Finally, three studies of this special issue were focused on development of antifouling systems following different approaches [35,36,37]. Francolini and colleagues [35] functionalized segmented polyurethanes, one of the most important class of biomedical polymers, with polyethylene glycol (PEG), known to possess strong antifouling properties. Findings showed how PEG-functionalization not only positively affected polyurethane ability to resist to *Staphylococcus epidermidis* adhesion but also improved mechanical properties of the polymer with clear advantages for practical applications. Cattò et al. [36] immobilized the protease α-Chymotrypsin, supposed to degrade the biofilm matrix, on a low-density polyethylene surface. Interestingly, enzyme immobilization significantly weakened *E. coli* biofilm formation affecting thickness, roughness, and surface area coverage but not bacterial viability, thus reducing the risk of drug resistance development. Finally, Faÿ and coworkers [37] developed antifouling paints by the use of three additives (Tween 80, Span 85, and PEG-silane) as surface modifiers. 

In conclusion, antimicrobial polymers may have a pivotal role in the global effort to find solutions against drug resistant infections. In the last 20 years, great scientific and technological advances have been made in this area, mainly thanks to the increased knowledge on mechanisms involved in materials/bacteria interaction as well as on the complexities of biofilm biology. Such knowledge was and still is the inspiration for biomaterials scientists to develop materials able to control biofilm formation. Despite that, a massive amount of work still remains to be done to address unsolved challenges, such as long-term stability, functionality, and biocompatibility of antimicrobial polymers. Translational research is also strongly needed in the near future, in order to make possible the transition of antimicrobial polymers from the bench to the patient bedside.
